# Overcoming Barriers to Human Papillomavirus Vaccination in Guangdong Province, China

**DOI:** 10.3390/vaccines13050482

**Published:** 2025-04-29

**Authors:** Shuaijing Zhang, Shiqi Li, Jingtai Ma, Guiyuan Ji, Zhifeng Li, Siyi Chen, Fenglin Zhang, Xingfen Yang, Jianpeng Xiao, Rong Cao, Chenggang Wu, Wei Wu

**Affiliations:** 1 Guangdong Provincial Institute of Public Health, Guangdong Provincial Center for Disease Control and Prevention, Guangzhou 511400, China; zhangshuaijing2001@163.com (S.Z.); lishiqi1999@126.com (S.L.); jingtaima@163.com (J.M.); jigy@gdiph.org.cn (G.J.); gdiph@cdcp.org.cn (Z.L.); kikichan@gdiph.org.cn (S.C.); jpengx@163.com (J.X.); caorongjsnt@126.com (R.C.); 2 NMPA Key Laboratory for Safety Evaluation of Cosmetics, Guangdong Provincial Key Laboratory of Tropical Disease Research, School of Public Health, Southern Medical University, Guangzhou 510515, China; xfyang@vip.163.com; 3 Guangzhou Institute of Microbiology Group Co., Ltd., Guangzhou 510700, China; zhangfenglin@gzswswyjs.wecom.work; 4 Guangdong Provincial Center for Disease Control and Prevention, Guangzhou 511400, China; 5 Zhuhai Center for Disease Control and Prevention, Zhuhai 519060, China

**Keywords:** HPV vaccine, HPV vaccination coverage, vaccination strategies, HPV genotype, school-based programs, male HPV burden, financial sustainability

## Abstract

Human papillomavirus (HPV) infection remains a critical public health challenge in China, particularly in Guangdong Province, where HPV-52, 16, and 58 genotypes predominate, and male infection rates exceed 40%. Despite the successful implementation of a government-funded school-based program that has achieved 88% HPV vaccine coverage among adolescent girls, several persistent barriers, including genotype mismatch (the free HPV vaccine covers < 50% of high-risk local strains), regional disparities (80% vs. 60% for first-dose coverage), and exclusion of males, thwart progress toward herd immunity. Financial sustainability risks pose an even more significant threat to the expansion of HPV vaccination programs, especially in Guangdong province where annual expenditures exceed CNY 200 million. This review delves into Guangdong’s pioneering efforts and proposes practical solutions: accelerating domestic multivalent HPV vaccine development, adopting gender-neutral vaccination policies, and leveraging mobile clinics for remote populations. These strategies not only provide a roadmap for China but also serve as valuable insight for other LMICs striving to overcome HPV-related inequalities.

## 1. Introduction

Human papillomavirus (HPV), a double-stranded DNA virus belonging to the family Papillomaviridae, comprises over 200 identified genotypes, approximately 40 of which specifically infect human anogenital epithelia [1]. Based on oncogenic potential, HPV genotypes are classified into two major categories: low-risk types (e.g., HPV-6, HPV-11), which are predominantly associated with benign mucocutaneous lesions including genital warts and recurrent respiratory papillomatosis; and high-risk types (notably HPV-16, HPV-18, HPV-31, HPV-52, HPV-58), which are strongly associated with various malignancies [2,3]. Epidemiological data reveal that HPV accounts for approximately 5% of the global cancer burden [4]. Cervical cancer constitutes 83% of HPV-associated malignancies, ranking as the fourth most common cancer among women worldwide [4,5]. Notably, anal cancer, the second most common HPV-associated malignancy, has shown a significant rise in age-standardized incidence rates over the past decade [6], underscoring the escalating public health challenge posed by HPV-related diseases.

Globally, the prevention and control of HPV-related diseases has become a public health priority. The World Health Organization (WHO) set forth the “90–70–90” targets to be achieved by 2030: 90% of girls fully vaccinated with HPV vaccine by age 15 years; 70% of women screened with a high-performance test by 35 years of age and again at age 45 years; and 90% of women identified with cervical cancer receive treatment [7]. However, there is still a large gap in HPV vaccination coverage in China. As of the latest reports, coverage among 15-year-old girls remains at only 10.15%, far lower than the 86% (95% CI: 71–89%) reported in countries such as Australia [8,9]. In response, domestic vaccine development has made great progress. In July 2024, the first domestically developed quadrivalent HPV vaccine, produced by the Chengdu Institute of Biological Products (CDIBP, Chengdu, China), was submitted for marketing approval—a milestone expected to enhance local supply, reduce costs, and improve nationwide HPV vaccine coverage.

In addition to the well-documented risks of cervical cancer in women, increasing attention must also be paid to HPV-related health risks among men. In recent years, the incidence of HPV-associated oropharyngeal cancer (OPC) has been rapidly increasing in the male population [10]. In Guangdong, the prevalence of HPV among men was 42.15% [11], exceeding the national average and highlighting the urgency of strengthening HPV prevention among males. As of 10 April 2025, a total of 76 countries or regions globally have implemented gender-neutral HPV vaccination strategies [12,13]. Although China’s national drug regulatory authority approved the use of HPV vaccines for males in January 2025 [14], current policies still primarily target females. This approach persists despite strong evidence that male vaccination can reduce oropharyngeal cancer incidence by up to 60% [15] and significantly enhance herd immunity [16].

Globally, HPV-16 and HPV-18 predominate and are strongly associated with most HPV-related cancers [17]. However, the East Asian region exhibits unique genotype distribution characteristics (e.g., Japan is dominated by HPV-52), suggesting that vaccine immunization strategies may need to be tailored to the different regions [18].

Sexual transmission represents the primary route of HPV dissemination, characterized by transient HPV Infection: approximately 90% of infections undergo spontaneous clearance within 2 years through host immune responses, demonstrating transient characteristics [1]. However, persistent high-risk HPV infection can induce epigenetic modifications in host cells, ultimately leading to malignant transformation at the infection site through the E6/E7 oncoprotein-mediated disruption of p53/Rb signaling pathways [19,20]. Consequently, the WHO advocates a three-tiered prevention strategy, with primary prevention focusing on HPV vaccination implementation and safe sexual behavior education.

Emerging studies suggest that HPV may also spread through non-sexual transmission routes. Environmental vectors such as contaminated medical instruments, textiles, and aqueous environments could facilitate viral dissemination, supported by the detection of fragmented HPV genomic components in untreated sewage [21,22]. However, the epidemiological significance of non-sexual transmission remains controversial, and its transmission efficiency has not yet been fully elucidated. This review focuses specifically on sexually transmitted HPV, while non-sexual transmission mechanisms are excluded from the current discussion scope.

This study collected publicly available data and literature related to HPV from three perspectives: epidemiological characteristics, vaccination coverage, and strategy optimization. By comparing international best practices with local implementation in Guangdong, this research provides actionable recommendations for optimizing HPV vaccination programs in Guangdong. Furthermore, the findings offer transferable strategies for low- and middle-income countries (LMICs) with similar health and economic profiles.

## 2. HPV Prevalence in Guangdong, China

HPV infection is globally prevalent, with an overall prevalence of 11.7% (95% CI: 11.6–11.7%) among women with normal cytology [23] and 31% (95% CI: 27–35%) among men [24]. HPV-16 and HPV-18 are the most oncogenic types globally [17], although genotype distribution varies by region; for example, HPV-52 is more prevalent in Asia [18].

Prior to the introduction of HPV vaccines in China, epidemiological survey data indicated that the overall HPV infection rate among Chinese women aged 20 years or older was 15% (95% CI: 14.1–15.9%) [25], which was notably higher than the global average (12%). This prevalence exhibited substantial regional variation and a bimodal age-specific distribution, with the first peak occurring in women aged 20–25 years (21.1%) and a second peak occurring in those aged 50–60 years (15.7%). The predominant HR-HPV genotypes were HPV-52 (3.5%), HPV-58 (2.1%), and HPV-16 (1.6%), underscoring the necessity for a population-based HPV vaccination strategy [25].

It is worth noting that the HR-HPV genotype distribution in China shows distinctive regional characteristics, with HPV-16, HPV-52, and HPV-58 constituting the main prevalent strains [26,27,28,29,30,31]. This genotypic heterogeneity underscores the importance of developing regionally tailored immunization strategies.

According to the most recent national cancer report, HPV-related malignancies impose a considerable disease burden in China. In 2022, the country recorded 119,300 new cases of cervical cancer (18.7% of the global total) and 37,200 deaths [32,33]. Moreover, the average annual growth rate of HPV-associated cancers in men reaches 3.7% (95% CI: 2.6–4.9%), with anal cancer showing the fastest increase at 7.5% (95% CI: 2.8–12.5%). Predictions indicate that the incidence of anal cancer among men in 2023 is expected to triple compared with 2015 levels [34]. As China’s most populous province, Guangdong Province faces a particularly severe HPV burden. Data from the provincial maternal and child healthcare institutions showed that the HPV infection rate among gynecological outpatients was 20.16% (95% CI: 19.98–20.33%), while the positive rate among those undergoing physical examination was 17.25% (95% CI: 16.58–17.94%) [35]. Among male outpatients, the infection rate reached as high as 42.15% [11], significantly surpassing the national average.

In Guangdong Province, HPV infections similarly display a bimodal age distribution and a unique pattern of HR-HPV genotype [36]. The province faces dual challenges in HPV prevention and control: First, persistent infections of HR-HPV types are characterized, with significantly higher infection rates of HPV-52 (6.8%), HPV-16 (5.2%), and HPV-58 (4.1%) than the other genotypes, and 12-month persistence infection rates of 67.3–72.8% [35,37]. Although HPV16 remains the most oncogenic, HPV52 and HPV58 are also high-risk types with confirmed carcinogenic potential and are frequently found in cervical cancer and precancerous lesions in China [37,38,39,40]. Second, although cervical cancer screening coverage has increased to 68.5%, the annual detection rate of cervical cancer continues to rise [41], indicating a need to further strengthen the existing prevention and control system.

## 3. HPV Vaccinations

HPV vaccination represents a primary and effective method of preventing HPV-related diseases and serves as the cornerstone of primary prevention against HPV-associated diseases. Currently, HPV vaccines are classified into three categories, including bivalent HPV vaccine (HPV-16 and -18), quadrivalent HPV vaccine (HPV-6, -11, -16, and -18), and nonavalent HPV vaccine (HPV-6, -11, -18, -31, -33, -45, -52, and -58).

Since 2016, five HPV vaccines have been approved for use in China (Table 1), including the imported quadrivalent vaccine (Gardasil^®^), nonavalent vaccine (Gardasil 9^®^), imported bivalent vaccine (Cervarix™), and two domestically produced bivalent vaccines (Cecolin^®^ and Walrinvax^®^) [42]. The domestic bivalent vaccines have captured 44% of the market share cost advantage, leading to a 62% reduction in per-dose costs compared to imported alternatives [43]. Nevertheless, the current domestic products do not adequately cover the locally prevalent HR-HPV types, specifically HPV-52 and HPV-58. Among the domestic multivalent vaccines under development, only three candidates, including a quadrivalent HPV-11/16/52/58 vaccine and a nonavalent HPV-6/11/16/18/31/33/45/52/58 vaccine, have progressed to Phase III clinical trials [44]. Although annual number of HPV vaccination doses administered and coverage rates have been steadily increasing, they remain below the global average and are insufficient to achieve herd immunity [45,46,47].

China currently has at least seven HPV vaccine candidates undergoing active clinical development. Domestic manufacturers are utilizing various expression platforms to develop multivalent vaccines, with several candidates in late-stage clinical trials and others in preclinical phases (Table 2). Notably, the quadrivalent vaccine developed by the Chengdu Institute of Biological Products (CDIBP, Chengdu, China) has already submitted a new drug application (NDA), suggesting imminent commercialization. This milestone may stimulate the competition in the Chinese HPV vaccine market, expanding vaccine access and reducing dependence on the imported vaccines. As regulatory milestones and clinical pipeline advancements accelerate, China’s HPV vaccine sector is positioned for transformative growth, which may substantially narrow the current accessibility gap in vaccination coverage. However, widespread market adoption will depend on three critical factors. First, cost-competitive pricing models aligned with domestic purchasing power. Second, sustained policy incentives to introduce HPV vaccines into the national immunization program. Last, public health efforts to promote vaccine uptake through awareness campaigns.

## 4. HPV Vaccination Coverage

As of the end of 2023, 143 of the 194 World Health Organization Member States had introduced HPV vaccines into their national immunization programs [8,51]. Coverage rates vary significantly by region, with the Americas and Europe achieving 85% and 77% coverage, respectively [8,51]. In contrast, LMICs lag significantly in the vaccine introduction and maintain coverage rates below one-third that of high-income countries [9]. According to the WHO 2024 health statistics report, the global estimate of (HPV) immunization coverage among 15-year-old girls was 15% [8], which was still far from the WHO target.

In 2022, the first-dose coverage rate of HPV vaccination among eligible Chinese females (9–45 years old) reached 10.15% [47]. However, marked age-related disparities were observed, with the lowest coverage (only 4%) in the key target group of girls aged 9–14 years old [47]. This rate falls significantly short of the levels reported in high-income countries [52]. Additionally, regional disparities are also pronounced. In economically developed cities such as Beijing and Shanghai, first-dose vaccination rates reached 25.40% and 20.99%, respectively [47]. Guangdong Province demonstrated a relatively high rate of 15.65%. In contrast, the rates of less developed areas such as Qinghai and Xinjiang were as low as 2.55% and 2.95%, respectively [47]. These disparities may be attributed to several factors, including economic development levels, access to healthcare resources, and public health awareness.

To address these challenges, China launched the Comprehensive Cervical Cancer Prevention and Control Strategy in 2021, starting with 15 pilot cities (districts). The strategy targets a 90% vaccination rate among 15-year-old girls by 2025 [53,54]. As of 2023, several provinces and cities in China, including Guangdong and Hainan, have successfully implemented free bivalent HPV vaccination targeting girls aged 13 to 14 years, achieving notable outcomes [48]. Pilot programs in many places have demonstrated impressive vaccination rates: the first-dose vaccination rate in Chengdu reached 90.04%, Jinan reached 94.4%, and Ordos reached approximately 70% [55]. Furthermore, the latest news from the National Health Commission reveals that the free HPV vaccination policy now covers about 40% of school-aged girls nationwide [55,56]. By adopting a two-dose vaccination schedule for the 9-to-14-year-old age female group, these initiatives have not only reduced the cost of vaccination but also significantly increased vaccine coverage, providing a solid foundation from which to further reduce the incidence of cervical cancer.

A study of cervical cancer prevention and control strategies implemented in Guangdong Province since September 2022 showed that vaccination coverage was significantly improved through a government-funded immunization program targeting girls under 14 years of age in the first grade of junior high school who were registered at schools in the province and had not previously been vaccinated against HPV [57,58]. As of January 2024, 656,000 cases (88.0% coverage) in the class of 2022 first-year girls’ cohort (*n* = 745,000) in Guangdong Province had completed the full course of vaccination, while the first-dose vaccination rate of the class of 2023 freshmen cohort (*n* = 788,000) was 81.0% (638,000 cases) [59]. Shenzhen’s practice serves as an example: a synergistic mechanism of “free vaccination provided by the government and supplementation at its own expense” had been put in place. By 2022, the target population’s first-dose vaccination coverage had risen to 82.1%. With the supplementation mechanism, predictive modeling suggests that the overall HPV vaccination rate for seventh-grade girls could reach 89% by the end of 2022, underscoring the effectiveness of this comprehensive approach. [60]. These findings are further supported by the latest data from Shenzhen in 2023. More than 92% of eligible seventh-grade girls completed HPV vaccination [61]. This real-world success not only strongly validates the forecasting model’s accuracy but also highlights the effectiveness of Shenzhen’s integrated strategy.

Nevertheless, persistent sub-province inequities in HPV vaccination coverage underscore systemic challenges across Guangdong Province. Fiscal subsidy allocation data reveal a stark divide: the developed areas within the Pearl River Delta (such as Guangzhou and Foshan) have achieved HPV vaccination rates of 80% or even higher. Conversely, the underdeveloped regions in western and northern Guangdong (including Maoming, Shaoguan, and Yunfu) have lower HPV vaccination rates, with some areas not having fully implemented free HPV vaccination, resulting in target population coverage of only 60–70% [62]. This 20-percentage-point disparity not only jeopardizes Guangdong’s 2030 90% coverage target but also mirrors vaccine access gaps observed in LMIC urban–rural divides.

Beyond structural and policy-level drivers, HPV vaccine uptake dynamics are shaped by a multi-ecological confluence of individual, social, and health system factors [63,64,65]. These include public awareness of HPV and its vaccine, perceived risk of infection, the accessibility of immunization services, cultural norms and stigmas, parental decision-making, and trust in healthcare providers. Vaccine acceptance is particularly correlated with educational level, household income, and urban versus rural residence [66,67,68,69,70]. These findings highlight the geographic disparities in HPV vaccination, necessitating the development of tailored, community-driven strategies to address localized barriers.

This study posits that subnational disparities in Guangdong Province emerge from three interrelated institutional factors: structural economic gradients, fragmented policy implementation, and sociocultural resistance. The affluent Pearl River Delta (PRD) region benefits from fiscal prioritization and early policy piloting, which allowed for campus activities and rapid resource mobilization. Conversely, less developed regions face delays in policy penetration due to limited fiscal autonomy. The sociocultural cleavage manifests as an urban–rural bifurcation: urban districts exhibit accelerated behavioral normalization of vaccination through multi-sectoral health communication initiatives, while rural areas exhibit high vaccine hesitancy due to misinformation and sociocognitive biases.

However, achieving high coverage relies heavily on sustained government financial investment, and ensuring long-term sustainability remains a challenge. Studies have shown that implementing a strategy that combines HPV vaccination with cervical cancer screening entails a cost of approximately CNY 152,000 per case of cervical cancer averted [71]. Although this approach is considerably more cost-effective than that of a single intervention, the pressure from projected enrollment growth (+2.7% per annum) [72] will lead to a steadily increasing annual budget requirement. Consequently, the demand for funding will exhibit a rigid upward trend.

The study revealed that the government-led immunization program boosted the willingness of women in the appropriate age group to receive the HPV vaccination. The number of non-immunization program HPV vaccine doses showed an exponential growth trend from 2020 to 2023, with the vaccine doses administered reaching 1.44 × 10⁶ doses in 2020, 3.47 × 10⁶ doses in 2021, 6.73 × 10⁶ doses in 2021, and 7.66 × 10⁶ doses in 2023 [73]. This remarkable growth trend suggests that the government’s continuous promotion of free HPV vaccination for 9- to 14-year-olds has effectively raised awareness and encouraged more women in the eligible age group to actively receive HPV vaccination.

## 5. HPV Vaccination Strategies

To achieve its targets, the WHO recommends prioritizing girls aged 9–14 years, who have not yet reached the age of sexual activity, as the primary target for HPV vaccination. In response, countries worldwide have adopted tailored strategies that align with their specific health system capacities and public health priorities, thereby supporting the global effort to eliminate cervical cancer.

China, as one of the countries bearing the heaviest HPV burden, has adopted a phased vaccination strategy marked by pilot programs and notable gender disparities. Although the HPV vaccine has been approved for female vaccination since 2016, it has not yet been included in the national immunization program. As a result, HPV vaccination remains largely voluntary and self-funded. On 8 January 2025, regulatory authorities approved the quadrivalent HPV vaccine for males aged 9 to 26 years to prevent anal cancer (caused by HPV-16 and -18) and genital warts (caused by HPV-6 and -11) [14]. This breakthrough marks a significant step forward in China’s HPV prevention strategy and is expected to reduce overall HPV transmission, accelerating the establishment of herd immunity [28,34].

In China, although the HPV vaccine has not yet been introduced into the National Immunization Program [13,45], several provinces have initiated free vaccination programs targeting school-age girls. Guangdong Province, in particular, has emerged as a national vanguard of HPV vaccination. Since September 2022, the provincial government has provided free HPV bivalent vaccinations to eligible girls under age 14 years. The program specifically targets girls who have registered for school in the province, are entering the first grade of junior high school, and have not previously received HPV vaccinations. The implementation of the program has been supported by the province’s financial resources, and it has achieved a vaccination coverage ranging from 81% to 88%. This high vaccination coverage has had a positive impact on the community, contributing to the prevention of HPV-related diseases. Looking ahead, Guangdong Province plans to expand its vaccination program to include women aged 15 and above. Additionally, the province also intends to establish integrated consortia for HPV vaccination counseling, cervical cancer screening, and related clinical services. These initiatives demonstrate Guangdong Province’s commitment to promoting HPV vaccination and improving women’s health.

Countries worldwide have adopted HPV vaccination strategies tailored to their health system capacities and public health priorities. In particular, developed countries such as the United Kingdom and Australia have achieved high vaccination coverage through school-based programs. The UK’s National Health Service (NHS) aims to eliminate cervical cancer by 2040 by offering free HPV vaccination to students aged 12–13, with the program extended to boys in 2019 [74]. Australia initiated its National HPV Vaccination Program in 2007, offering free vaccines to females aged 12–26 and providing catch-up doses through school and community-based services [75,76]. In contrast, Japan suspended active HPV vaccine recommendation in 2013 but resumed it in 2022 with a three-year catch-up program [77]. In 2023, it introduced the nonavalent vaccine into its National Immunization Program, indicating a strengthened commitment to HPV prevention. Meanwhile, LMICs, such as Rwanda, South Africa, Mexico, and Brazil, have made significant progress through partnerships with multilateral organizations like Gavi and by leveraging school-based delivery models, despite limited resources [73,74,75,76]. Research indicates that over 90% of HPV vaccination programs in LMICs rely on school-based platforms, which are particularly effective in resource-constrained settings [78].

In summary, countries across different income levels have adopted context-specific strategies to implement HPV vaccination programs, tailored to their healthcare infrastructure and financial capacity. Experiences from Rwanda, Brazil, and Guangdong provinces in China highlight the critical role of political commitment, innovative financing mechanisms, and integration with existing health infrastructures. These international practices provide valuable insights for other regions, particularly LMICs, seeking to further improve HPV vaccine uptake and optimize implementation strategies.

## 6. Challenges and Recommendations

### 6.1. Challenges

Despite substantial progress, HPV vaccination programs in China still face several critical challenges.

- Scientific Fact: The free HPV vaccine genotype mismatch limits vaccine effectiveness

HPV vaccines approved for use globally include bivalent, quadrivalent, and nonavalent vaccines. Notably, HR-HPV genotypes HPV-52 and HPV-58, which are particularly prevalent in China and accounting for 26% of persistent infections, are exclusively covered by the nonavalent vaccine [36]. Only two domestically produced bivalent vaccines are available in the Chinese market, while other multivalent vaccines remain under development. The existing accessibility and choice of vaccines present certain constraints [43,79,80], making it challenging to fulfill the substantial domestic demand for vaccination solely through imported vaccines.

- Operational Gap: School-based programs fail to reach some target population

School-based HPV vaccination programs alone are insufficient to reach all eligible adolescents. The junior high attendance rate in rural Guangdong is not 100%, standing at 96.64% [81], meaning a portion of the age-appropriate vaccination population does not receive free HPV vaccination. Furthermore, due to the lack of outreach strategies for out-of-school populations, the coverage gap continues to widen, ultimately hindering the development of herd immunity.

- Financial Constraint: Over-reliance on Provincial Funding Jeopardizes Program Stability

The HPV vaccination program in Guangdong Province relies heavily on local financial resources, posing a significant risk to its long-term sustainability. Despite a 300% increase in the HPV vaccine budget since 2022 [59], the program still only covers 80–81% of the target population. Without an infusion of national financial support or collaboration with private partners, the vaccine program faces the risk of stagnation or even potential disruption.

Subnational governments have limited market influence, and vaccine procurement—conducted independently at the provincial and municipal levels—results in high prices ranging from USD 16 to USD 180 per dose, significantly exceeding the median prices secured by some organizations like Gavi [82].

- Sociocultural Factors: Misconceptions linking vaccination to promiscuity reduce male uptake

Sociocultural factors significantly contribute to HPV vaccine hesitancy. The limited accessibility and impact of HPV-related health education have led to persistent misconceptions, including a preference for high-valency vaccines, delays in HPV vaccination due to nonavalent vaccine shortages, and concerns about vaccine costs [63,83,84]. Among males, structural barriers arise from gendered public health narratives linking HPV only to cervical cancer, ignoring its role in male-associated cancers [85]. Additionally, stigma associating HPV vaccination with promiscuity further exacerbates male hesitancy [86]. These misperceptions are reflected in very low male vaccination coverage in Guangdong.

### 6.2. Recommendations

First, it is recommended that the process of introducing the HPV vaccine into the National Immunization Program should be accelerated and that a gender-neutral immunization strategy be established simultaneously [87]. Health economics evidence suggests that including males in HPV vaccination not only yields significant cost-effectiveness advantages but also contributes to stronger herd immunity and prevents cross-infection of HPV [83,88]. In parallel, efforts should be made to advance the development of new multivalent vaccines covering more prevalent genotypes and to optimize vaccination schedules, thereby improving the efficiency of HPV prevention and control.

Second, to improve vaccination coverage among school-age girls, it is recommended that HPV vaccination should be made a requirement for school enrollment, drawing on Shenzhen’s policy of “no vaccination, no school enrolment”. To further expand coverage, the restrictions based on school or residential registration should be gradually removed, allowing access to free HPV vaccination for girls who have dropped out of school and children from migrant families. Initially, the program could prioritize schoolchildren currently enrolled in school and later be extended to all school-age girls at community vaccination clinics, regardless of educational or residential documentation.

Third, Guangdong province should explore diversified financing modes to alleviate fiscal pressure. One approach is to establish a multi-channel financing mechanism, promote the “government + enterprise” risk-sharing model, and sign long-term procurement agreements with domestic enterprises such as Wantai Biologicals drawing on Rwandan’s experience [89]. This strategy could offer tax incentives in exchange for reduced vaccine prices. Another approach is to implement a precise subsidy policy, providing additional subsidies for HPV vaccines supplied to the eastern, western, and northern regions of Guangdong, and to set up a weighted scoring mechanism for the supply of less developed regions in the procurement bidding to narrow the regional vaccination gap. A third option is to build a long-term protection framework by including HPV vaccines under public health specialties or health insurance reimbursement—similar to the “first shot free” model in Toketo County, Inner Mongolia, and Zhejiang’s use of personal health insurance accounts [90]. Through the three-phase strategy of “short-term cost reduction, medium-term equalization, and long-term stabilization”, financial inputs have increased, and vaccination services have been sustainably covered. Studies indicate that Guangdong’s volume-based procurement strategy, which reduces vaccine prices by 20% and achieves 90% vaccination coverage in the target population, is highly favorable in terms of cost-effectiveness (incremental cost-effectiveness ratio/gross domestic product (ICER/GDP) < 0.5) [91].

Fourth, correcting persistent misinformation about HPV vaccination requires an institutionalized, multi-level intervention integrating school education, community engagement, and health promotion. School-based initiatives, such as incorporating HPV-related content into the curricula and providing on-site vaccination services, have been shown to significantly increase vaccination acceptance among adolescents and their parents [63]. At the community level, mobilizing grassroots health institutions and local organizations can enhance vaccination literacy, especially in rural and underserved areas [92]. Additionally, consistent public outreach through expert-led seminars and multimedia campaigns can help dispel misinformation and improve HPV vaccination uptake. Evidence suggests that interventions, including school-based education and vaccination efforts, are cost-effective and can lead to significant improvements in vaccine uptake [93]. A multi-level, cross-sectoral collaboration framework is essential to overcome the structural and sociocultural barriers to improve vaccine uptake.

Fifth, deploying mobile vaccination clinics is a practical and cost-effective strategy to expand HPV vaccination coverage in remote and underserved areas. This approach, modeled on Inner Mongolia’s mobile vaccination vehicles, has proven effective in increasing vaccination coverage in hard-to-reach pastoral regions. Similar methods have also been employed in the United States for other vaccination programs [94]. Research shows that mobile services significantly reduce geographic and socioeconomic barriers, enhancing immunization equity [95]. Mobile vaccination is also highly cost-effective in various settings [96]. Therefore, integrating mobile clinics into China’s national HPV immunization program, supported by local financial incentives and cross-sectoral cooperation, can provide an equitable and scalable solution for rural populations.

Finally, the importance of long-term strategic interventions must be emphasized. Key measures are as follows: (1) establishing a national HPV vaccination fund through a government–corporate social responsibility (CSR) partnership, drawing on the public–private partnership framework of the NHS; (2) developing artificial intelligence-driven platforms for real-time monitoring of vaccination coverage and optimizing the supply chain (refer to the smart tracking methodology used by the U.S. Centers for Disease Control and Prevention); (3) implementing a pilot HPV vaccination program targeting the male population. For instance, Australia’s school-based program post-2013 excluded 84% of eligible males due to age limits [97]. This gap underscores the need for community-based vaccination alongside school initiatives. In China, pilot initiatives could prioritize high-risk male groups in industrial hubs, such as migrant workers in Guangzhou, where HPV-16-driven oropharyngeal cancer is increasingly prevalent.

## 7. Conclusions

Guangdong’s HPV control journey highlights a vital lesson: achieving high HPV vaccination coverage requires more than just providing free vaccines, it also requires genotype-specific products, inclusive policies, and innovative financing strategies. The province’s achievement in vaccinating 88% of schoolgirls demonstrates the effectiveness of centralized governance. However, its challenges with male vaccination uptake and access in rural areas reflect global inequities. To accelerate HPV control in China and contribute to the WHO’s 2030 elimination goals, immediate actions are needed. Specifically, we recommend the following:

- Accelerating the regulatory approval of domestically produced multivalent HPV vaccines to expand HPV vaccine access and reduce dependency on imported products.
- Enacting gender-neutral vaccination mandates to promote equity and extend protection to all adolescents, regardless of gender.
- Harnessing digital technologies to optimize vaccine distribution and enable cross-regional coordination, particularly for underserved rural areas.

These recommendations are practical, timely, and aligned with the evolving needs of both domestic and global public health agendas. Guangdong’s experience, characterized by collaboration, financial subsidies, and public health education, provides valuable insights for regions, especially LMICs, that are looking to scale up HPV vaccination coverage. As China strengthens its role in global health governance, the lessons learned from Guangdong’s approach can serve as a scalable, equity-driven model for HPV vaccination strategies in other LMICs, contributing to the global effort to eliminate cervical cancer.

## Figures and Tables

**Table 1:** Prophylactic HPV vaccine in China, 2024 [48,49].

Trade Name	Manufacturer	Antigens (VLP Types)	Licensing Year	Approved Age	Market Share (%)
Cervarix™	GSK	HPV-16, HPV-18	Approved in 2016	Female aged 9–45 years	3.6
Gardasil^®^	Merck	HPV-6, HPV-11, HPV-16, HPV-18	Approved in 2017	Female aged 9–45 years and male aged 9–26 years	24.0
Gardasil 9^®^	Merck	HPV-6, HPV-11, HPV-16, HPV-18, HPV-31, HPV-33, HPV-45, HPV-52, HPV-58	Approved in 2018	Female aged 9–45 years	25.0
Cecolin^®^	Xiamen Innovax	HPV-16, HPV-18	Approved in 2019	Female aged 9–45 years	3.4
Walrinvax^®^	Shanghai Zerum (Walvax)	HPV-16, HPV-18	Approved in 2022	Female aged 9–30 years	44.0

GSK Glaxo Smith Kline Plc, London, United Kingdom; Xiamen Innovax Xiamen Innovax Biotech Co., Ltd., Xiamen, Fujian, China; Ze-run (Walvx) Shanghai Zerun Biotechnology Co., Ltd. (Walvx), Shanghai, China.

**Table 2:** Prophylactic HPV vaccine development in China, 2024 [50].

Manufacturer	Antigens (VLP Types)	Expression Platform	Phase of R & D
Sinocelltech	HPV-14 (6,11,16,18,31,33,35,39,45,51,52,56,58,59)	Insect cells	Phase III
CNBG	HPV-11 (6,11,16,18,31,33,45,52,58,59,68)	*Hansenula polymorpha*	Phase III
CDIBP	HPV-4 (6,11,16,18)	*Hansenula polymorpha*	NDA Submitted
Shanghai Bovax	HPV-15 (6,11,16,18,31,33,35,39,45,51,52,56,58,59,68)	*Hansenula polymorpha*	Phase II
Liaoning Bovax	HPV-15 (6,11,16,18,31,33,35,39,45,51,52,56,58,59,68)	*E. coli*	Phase II
Xiamen Innovax	HPV-9 (6,11,16,18,31,33,45,52,58)	*E. coli*	Phase II
Stemirna Bovax	HPV-9 (6,11,16,18,31,33,45,52,58)	*Pichia pastoris*	Phase III

Sinocelltech Beijing Sinocelltech Group Co., Ltd, Beijing, China.; CNBG Biological Technology Institute, Beijing, China; CDIBP Chengdu Institute of Biological Products Co., Ltd, Chengdu, China.; Shanghai Bovax Shanghai Bovax Biotechnology Co., Ltd., Shanghai, China; Liaoning Bovax Liaoning Chengda Biotechnology Co. Ltd, Shenyang, China.; Xiamen Innovax Xiamen Innovax Biotech Co., Ltd., Xiamen, China; Stemirna Therapeutics Co., Ltd., Shanghai, China.

## Data Availability

No new data were created or analyzed in this study. Data sharing is not applicable to this article.

## Abbreviations

The following abbreviations are used in this manuscript:

HPV	Human papillomavirus
WHO	World Health Organization
LMICs	Low- and middle-income countries
DNA	Deoxyribonucleic acid
OPC	Oropharyngeal cancer
HR-HPV	High-risk HPV
GSK	Glaxo Smith Kline Plc
Xiamen Innovax	Xiamen Innovax Biotech Co., Ltd.
Ze-run (Walvx)	Shanghai Zerun Biotechnology Co., Ltd. (Walvx)
NHS	National Health Service [32]
